# How Does Metro Maintenance Staff’s Risk Perception Influence Safety Citizenship Behavior—The Mediating Role of Safety Attitude

**DOI:** 10.3390/ijerph18105466

**Published:** 2021-05-20

**Authors:** Huaiyuan Zhai, Mengjie Li, Shengyue Hao, Mingli Chen, Lingchen Kong

**Affiliations:** 1 School of Economics and Management, Beijing Jiaotong University, Beijing 100044, China; hyzhai@bjtu.edu.cn (H.Z.); haoshyue@bjtu.edu.cn (S.H.); chenml@bjtu.edu.cn (M.C.); 2 School of Economics and Management, Cangzhou Jiaotong College, Cangzhou 061199, China; 3 School of Science, Beijing Jiaotong University, Beijing 100044, China; lchkong@bjtu.edu.cn

**Keywords:** metro maintenance staff, risk perception, safety citizenship behavior, mediating role

## Abstract

The accident rate is high in subway maintenance work, and most of the accidents are caused by human factors, especially the lack of sensitivity to risk perception, the lack of rigorous attitude towards safety and the lack of safe citizenship behavior (SCB). Therefore, it is very important to study the risk perception (RP), safety attitude (SA) and SCB of metro maintenance staff in order to reduce the accident rate. In order to reduce human errors and accidents, this study analyzed the influence of metro maintenance staff’s RP on their SCB and the mediating role of SA. Based on previous studies, this paper uses the risk perception scale, safety attitude scale and safety citizenship behavior scale as research tools. A survey was administered at the Subway Company, and altogether 268 valid questionnaires were used, and the data were analyzed by SPSS19.0 (IBM, Armonk, NY, USA) and AMOS 24.0 (IBM, Armonk, New York, NY, USA). The result reveals that SA plays a complete mediating role between metro maintenance staff’s RP and their SCB; and SA has a positive influence on SCB; RP has a positive influence on SA; and SA positively predicts SCB.

## 1. Introduction

The development of subway and light railway (SL) keeps its pace with the fast expansion of cities in China. The incidents associated with SL are also increasing rapidly. For example, on 23 June 2010, a major safety accident occurred in Beijing Metro when an employee fell from the top of the car and died after rescue. Human error is the most difficult factor to predict of all the factors in the SL operation. Xiong [1] reveals that more than 70% of incidents are caused by human mistakes. Human error is the bottleneck of the SL safety operation. Lund and Aaro put forward that accidents can be prevented; we should change our attitude towards safety first and then change our own safety behavior, and finally reduce the probability of accidents [2]. Consequently, there is important significance to analyzing the safety behavior of metro maintenance staff, especially safety citizenship behavior (SCB) of metro maintenance staff.

Previous research on SL safety issues concentrates on three aspects: the influence factors on safety behavior, the relationship between risk perception and risk behavior [3,4], and the correlation of safety attitude and risk action [5,6]. Mearns and Flin [7] proposed that safety attitude, safety behavior, safety perception and risk evaluation are the key factors to prevent SL incidents. Liu et al. [8] proposed that among the factors of injury prevention and improvement of workplace safety, safe citizenship behavior (SCB) is an important one. Namian et al. [9] believed that only by recognizing the hazards and risks of work can we effectively reduce workplace accidents. Arezes, P.M. [10] showed that their safety behavior could be predicted by personal risk perception and other perceived cognitive factors. Based on previous research, catastrophic safety accidents are often caused by poor hazard identification and underestimation of safety risks [11], and we can improve safety behavior by increasing risk perception [12]. However, there is limited research on the relationship among safety citizenship behavior (SCB), risk perception (RP) and safety attitude (SA). In particular, the intermediary role of SA between RP and SCB is rarely mentioned. As such, this study focuses on the human factor of SL metro maintenance staff using structural equation model (SEM) to analyze the relationship between SCB, RP, and SA, and focuses on the mediating role of SA.

## 2. Literature Review

### 2.1. Risk Perception

Risk is a highly interdisciplinary concept [13]. Risk perception (RP) can be referred to as the intuitive risk judgment made by the majority of people to evaluate hazards [14]. It is the attitude of perception to risk factors, the probability of the risk occurrence and the potential damage when it happens [6]. Tränkle, Gelau, & Metker [15] put forward that risk perception is a kind of risk situational awareness, which refers to the subjective cognition and evaluation of the potential danger of the external environment, as well as the corresponding preparatory behavior. This has been affected by the individual’s sensitivity to the surroundings and his or her alertness to the potential risk. Ramsey and Rickson [16] proposed that people had five reaction steps to potential risks: risk perception, risk acknowledgement, decision on avoidance, ability of avoidance and safety behavior. Incidents arise whenever one of the five steps fails. Underestimating surroundings and overestimating individual reaction ability leads to a misleading risk perception, and thereafter the behavior to avoid incidents. Risk perception has been extensively studied in many fields. Some public health-related issues, such as binge drinking [17,18], chicken contamination [19] and influenza vaccination [20]; and some occupational safety issues, such as the usage of hearing protection devices [10], involvement in safety management [21] and the aviation industry [22,23] are all introducing a lot of research on risk perception. Similarly, in the field of construction safety [24,25], risk perception has been well studied. In addition, An-Magritt Kummeneje [26] found that risk perception is one of the harbingers of conflict with other road users while riding. Xia et al. [27] believes that risk perception may have a negative or positive impact on safety behavior, which mainly depends on the environment and can be regarded as a work obstacle or a job challenge.

Generally, the two parts of cognition and emotion together constitute risk perception [28,29]. Colin [30] believed that risk perception includes four dimensions: risk augmentation, dread, collective vulnerability and knowledge. Lifshitz [31] proposed that risk perception is a two-factor structure composed of later-life risks and terror risks. Rundmo [32] thought that an important factor in judging risk perception is the degree of worry and whether employees feel safe or unsafe. Therefore, combined with the above viewpoints and the characteristics of metro maintenance staff, this paper divides risk perception into three dimensions, in line with Man et al. [33]: risk perception—severity, risk perception—worry and risk perception—safety.

### 2.2. Safety Attitude

Attitude is one of the most important research topics in psychology. Seaboch [34] concluded that attitude is the most important aspect in order to understand a person’s behavior. The definition of attitude from Oppenheim [35] is the reaction or response resulting from an individual’s characteristics or personality, which can also be understood as a person’s safety attitude deciding his or her safety behavior. It has been proven that safety attitude (SA) can effectively improve staff safety. Although SA is a mental activity, it can directly affect and dominate people’s behavior [36]. SA can reflect the general situation of employees’ safety work, help employees realize the importance of safety policies, and then ensure the smooth implementation of safety policies and promote the real implementation of safety regulations [37,38]. Previous research reveals that incidents are caused by incorrect response to a situation, which results from incorrect SA towards a potentially dangerous situation. Heinrich [39] suggested that the most important aspect that leads to one’s unsafe behavior is his or her attitude. Monazzam and Soltanzadeh [40] discovered that workers who are too optimistic and believe that accidents will not happen are more likely to be at risk and injured. Li, et al. [41] found that the motorcycle drivers’ SA decides their road manners. Li and Huang [42] also found SA is significantly related to the ratio of incidents. Rau [43] proposed that SA can effectively predict traffic accidents and workplace accidents.

For safety attitude (SA), Cox [44] initially proposed that SA should be divided into five dimensions: personal skepticism, individual responsibility, safeness of work environment, effectiveness of arrangements for safety and personal immunity. Rosenberg and Hovland [45] divided attitudes into three dimensions: safety awareness, safety behavior tendency and safety emotion. Sexton et al. [46] and Loosemore [47] believed that the SA scale consists of five dimensions: teamwork atmosphere, safety atmosphere, management concept, job satisfaction and working conditions. White et al. [48] thought that SA should be measured in terms of five dimensions: advantages, disadvantages, referents, barriers and facilitators of safety adherence. Therefore, combined with previous research results and the characteristics of subway maintenance personnel, SA is divided into three dimensions: safety awareness, safety emotion and safety intention.

### 2.3. Safety Citizenship Behavior

Hofmann et al. [49,50] formally put forward the concept of safety citizenship behavior (SCB) for the first time, namely, the voluntary behavior of construction personnel to ensure the safe performance of other team members and achieve the safety goals of the project and organization. It was found that SCB is very important for improving the safety performance of working groups and emphasizing mutual support among employees so as to improve organizational efficiency. Based on the above, Shama et al. [51] further defined SCB as spontaneous helping of other team members to improve safety behavior with behaviors beyond their scope of work, which further proved that SCB was an important factor to ensure the safety of working groups [52,53]. Through the research, it is found that the main reason for construction accidents is the unsafe behaviors of workers, and one of the important means to reduce accidents is to increase the safety behaviors in the group [54,55]. Therefore, it is of great significance to study the factors affecting SCB and then actively intervene and increase the safety behavior of employees and reduce the occurrence of accidents. This study focuses on understanding how RP affects SCB through the role of SA, which has high research value.

While studying the relationship between leader–member exchange and safety citizenship behavior (SCB), Hofmann proposed that SCB is a multidimensional construct composed of helping colleagues, safe proposal, responsibility awareness, safe communication, civic ethics and spontaneous change [49,50]. In the context of the chemical industry, Curcuruto and Griffin [53] proposed a SCB model with four dimensions: safety stewardship, affective commitment, safety voice and psychological ownership. Liu et al. [8] further proposed that SCB should be divided into four dimensions: safety proposal, responsibility awareness, active participation and helping colleagues. In this paper, considering the complexity of railway workers’ job characteristics and current safety regulations, SCB is divided into four dimensions, in line with Meng et al. [56], with improvements: help colleagues, suggestions, whistleblowing and safe civic virtues.

### 2.4. Dimensions

In the construction of the latent variable dimension, we must consider the job characteristics of subway staff: subway maintenance personnel often work at night, complete live line work and group work, and the working environment is complex and has more collaborative work. Combined with the literature analysis of 2.1, 2.2 and 2.3 above, the dimensions and abbreviations of RP, SA and SCB are shown in Table 1 below.

### 2.5. Research Hypotheses

As the model of preventive adoption process proposed by Westin points out, the emergence of behavior must go through seven stages: not aware of the problem, aware of the problem but not personally involved, participating and deciding what to do, planning to take action but have not yet taken action, deciding not to take action, taking action and maintaining action [57]. The process from “not aware” to “participating and deciding what to do” is the process from perceiving risk to forming an attitude when facing risk. Rickson [16] et al. also proposed that there are five steps for people to respond to potential risks: risk perception, risk cognition, avoidance decision, avoidance ability and safety behavior. When faced with a risk, employees first form the perception of the risk, then form the awareness of the risk and then build their own safety attitude, resulting in personal judgment. The difference of risk perception level will lead to the formation of different SA [58]. The stronger the perception of risk, the deeper the awareness of safety, the more likely they are to take countermeasures [59].

Therefore, the following hypothesis H1 was proposed: RP has a positive impact on SA.

The relevant research shows that the SA of workers has a direct relationship with safety behavior [60]. The SA has a significant impact on safety behavior [61], especially the attitude of employees on accident prevention having a significant impact on their behavior safety at the individual and organizational level [62]. Meng et al. [56] further proposed that increasing safety concerns and workers’ attitudes play a positive role in promoting SCB. Li et al. [63] also proposed that SA has a positive impact on the SCB.

Therefore, the following hypothesis H2 was proposed: SA has a positive impact on SCB.

Many studies have also mentioned that risk perception has a positive impact on safety behavior [64], especially if there is a significant positive correlation between risk perception and phase related to self-protection [65]. Xia et al. posit that employees’ risk perception varies as a job hindrance or challenge in different contexts, which then decreases or increases safety behavior in different population samples from different occupations, respectively [27].

Therefore, the following hypothesis H3 was proposed: RP has a positive impact on SCB.

As mentioned above, it can be concluded from previous research and the data of China’s SL industry that there is a positive correlation between RP, SCB and SA. 

We also propose hypothesis H4: SA mediates the relationship between RP and SCB.

**Figure 1:**
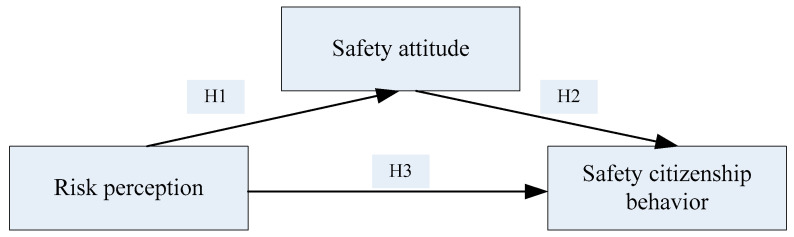
Framework of the influence model and the hypotheses among safety citizenship behavior, safety attitude and risk perception.

All the hypothetical relationships are shown in Figure 1 below.

## 3. Method and Data Analysis

### 3.1. Quantitative Scale

This scales first counts information on the respondents’ socio-economic background. It includes gender, age, education level and years of work experience. On the basis of the existing questionnaire, we revised it according to the characteristics of metro maintenance work, and obtained the questionnaire used in this study.

For RP scales, we followed the scales in Man, et al. [33], and revised 22 items. We used the Likert scale 7 and divided each question into 7 levels, from unsafe to not at all severe, which are represented by 7, 6, 5, 4, 3, 2, 1, respectively. The SA scale used in this study follows the attitude to safety questionnaire in Seaboch [34] and the safety attitudes scale in Cox [44], and was revised to 14 items. Each question of each part has seven options that represent: totally agree, somewhat agree, uncertain, somewhat disagree and totally disagree, which were assigned the following values: 7, 6, 5, 4, 3, 2, 1. The SCB scales followed the SCB questionnaire in Meng [56]. The SCB scale has 33 questions and questions in this part adopt the same seven-level evaluation system as the one in the above-mentioned SA scale.

All of the scales were adapted to take into consideration the special operational requirements of the metro maintenance staff. The total grade that a respondent received from each part represents his or her level. The higher the score was, the better the respondents had a more sensitive RP, a better SA and a better SCB. Please refer to Appendix A for details of these questionnaires.

### 3.2. Participants and Procedure

In this study, we used questionnaires as a tool to verify our hypotheses. The questionnaire was distributed to more than 300 metro maintenance staff with a cover letter which provided the instructions to explain the purpose of this study and to direct respondents to answer our questions. The responding rate was 100%, with 300 questionnaires returned. After excluding questionnaires with missing and invalid information, 268 valid responses were collected; this corresponds to 89.3% of the total questionnaire distribution. Among them, a large number of questionnaires were excluded because the respondents only filled in one or two of the three questionnaires, which did not meet the requirements for the integrity of the questionnaires.

### 3.3. Data Analysis Procedures

This paper combs the ideas and steps of data analysis here. SPSS 19.0 (IBM, Armonk, NY, USA) and AMOS 24.0 (IBM, Armonk, New York, NY, USA) were used for data processing and statistical analysis. The specific steps are as follows:

(1) Descriptive statistical analysis, which describes basic information of sample;
(2) KMO and Bartlett sphericity test to test the validity of the data [66];
(3) Cronbach’s alpha to test the reliability of the scale [67,68];
(4) Parameter significance, convergence and discriminant validity analysis;
(5) Structural equation modeling technique to test hypotheses;
(6) Bootstrapping method to test the significance of indirect effects (2000 samples, interval of confidence: 0.95) [69];
(7) According to the results of data analysis, specific improvement measures are considered.

### 3.4. Descriptive Statistics

As shown in Table 2, among the 268 respondents, male workers dominated the occupation, with 73.1%. The workers were relatively young and at an early period of their career, with most of them are between 26 and 40 years old. In total, 83.2% of the sample worked more than five years. The majority of the sample did not have bachelor’s degree; 62.7% were at the college, high school or lower education level.

### 3.5. Data Validity

The questionnaire was tested using SPSS19.0, and KMO and Bartlett sphericity tests were performed, as shown in Table 3. The results showed that the KMO value of the RP scales were 0.958, the KMO value of the SA scales were 0.909, the KMO value of the SCB scales were 0.966, and the KMO value of all the data were 0.961, which were all more than 0.7. The significant value of these scales were all less than 0.05. All the above facts proved that all variables were significant at the level of 0.05 and that the data were valid and suitable for factor analysis [66].

### 3.6. Reliability Analysis

The Cronbach’s coefficient was used to measure the reliability of the questionnaire. In order to ensure the reliability of the data, it is recommended to use a Cronbach’s alpha value higher than 0.70 [70]. SPSS 19.0 was used to analyze the reliability of RP, SA and SCB. The results are shown in Table 4 below.

It can be seen from Table 4 that the Cronbach’s alphas of RP, SA and SCB were all greater than 0.7. After deleting each item, the alpha value of each measurement model was less than the alpha value of the initial measurement model. Therefore, the items of RP, SA and SCB had good reliability, namely the RP was 0.866, SA was 0.729 and SCB was 0.872, which were all greater than the Cronbach’s alpha value of the deleted item. Except for SA1, SA2 and SA3, the total correlation coefficient (CITC) of each measurement item of each construct was greater than 0.7, showing a strong reliability; SA1 and SA2 were only greater than 0.5, SA3 was only greater than 0.6, indicating that the reliability of SA1, SA2 and SA3 was slightly worse, but still met the requirements of data reliability.

### 3.7. Convergence Validity Analysis and Discriminant Validity Analysis

This paper used AMOS 26.0 to analyze the significance and convergence effectiveness of each safety construct parameter, and the results are shown in Table 5.

It can be seen from Table 5 that the standardized estimate (Std) of each observation variable was greater than 0.6, the average variance extracted (AVE) of each latent variable was greater than 0.5 and the composite reliability (CR) was greater than 0.6, so they were verified to have high convergence validity. All factor loads were greater than 0.6 and all squared multiple correlations (SMCs) were greater than 0.36, except two that were 0.37 and 0.539, respectively. The others were all greater than 0.6, showing that the reliability of all subjects was good. All *p* values were less than 0.001, indicating the results were significant [71].

The discriminant validity test results between RP, SA and SCB are shown in Table 6 below. It can be seen from Table 6 that the discriminant validities between SCB, SA and RP were moderate but acceptable. From the perspective of SCB, 0.817 is greater than 0.164 but less than 0.876, so the discriminant validity between SCB and RP was good; the discriminant validity between SCB and SA was not obvious but acceptable. From the perspective of SA, 0.713 is less than 0.876 but more than 0.217, so the discriminant validity between SA and SCB was not obvious but acceptable, and the discriminant validity between SA and RP was good. From the perspective of RP, 0.839 is greater than 0.164 and 0.217, so the discriminant validities between RP and SCB, RP and SA were good. In summary, the discriminant validity of RP, SA and SCB was acceptable [72].

## 4. Results

### 4.1. Hypothesis Test

#### 4.1.1. Intermediary Effect Analysis Procedure

In order to verify the mediating role of SA, we mainly drew lessons from Wen’s [73] intermediary verification procedure:

(1) Test the influence coefficient c of RP on SCB in Model 1. If it is significant, there is a mediating effect, otherwise it will have a masking effect. Regardless of whether it is significant or not, follow-up tests are performed.
(2) Test the influence coefficient a of RP on SA and the influence coefficient b of SA on SCB in turn in Model 2. If the two coefficients tested above are both significant, the indirect effect is significant. Turn to Step 4. If at least one of them is not significant, proceed to Step 3.
(3) Use the Boostrap method to directly test H_0_: ab = 0. If it is significant, the indirect effect is significant and proceeds to Step 4, otherwise the indirect effect is not significant and the analysis should be stopped.
(4) In test Model 2, if the influence coefficient c’ of RP on SCB is not significant, that is, the direct effect is not significant, this indicates that SA is completely intermediary. If it is significant, the direct effect is significant. Go to Step 5.
(5) Compare the signs of ab and c’. If the signs are the same, they are part of the mediation, and the mediation effect accounts for the ab/c of the total effect. If the different signs are the cover effect, report the absolute value of the ratio of the indirect effect to the direct effect|ab/c’|.

#### 4.1.2. Data Analysis

(1) In order to test the fitness of the scales, AMOS was used. In general, the following values and thresholds for each test are considered as significant: the value of chi-square (χ^2^/df) is between 1 and 3; the values of GFI and CFI are greater than 0.9 and the closer to 1, the better; the values of RMR, on the contrary, with a threshold 0.05, and the value of RMSEA is less than 0.08 [74], if they are all closer to 0, the better [71,72,75,76], just as Table 7 shown.

**Figure 2:**
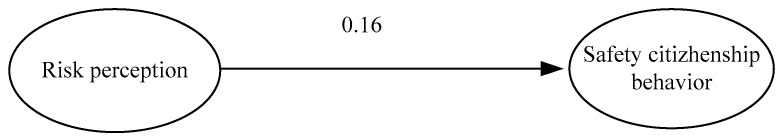
The results of model M1.

We first built the M1 model to estimate the indirect affection between RP and SCB(c), with all the values of fitting scales as shown in Figure 2 and Table 8. The chi-square estimate in the model was 1.503. RMR and RMSEA values were less than 0.05, which reveals the results are significant. The estimates for GFI, CFI and IFI were all greater than 0.9 and close to 1, which proves the model M1 the paper adopted fit the data well. Hypothesis H3 was confirmed: RP has a positive impact on SCB. The standardized estimate factor loading was 0.16 and was significant at the 0.05 level (*p* value was 0.02). All of these prove the existence of mediation.

**Figure 3:**
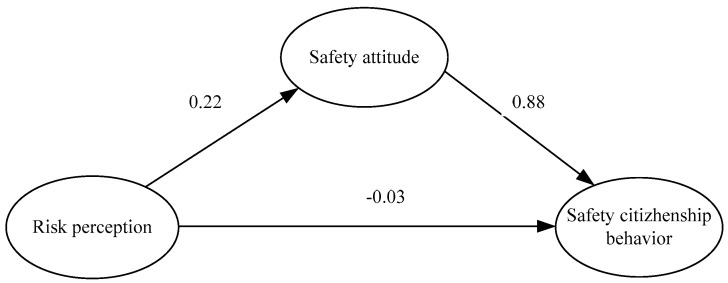
The standardized results of model M2.

(2) In mode M2, as shown in Figure 3 and Table 9, the standardized coefficient from RP to SCB(c’) was equal to −0.03 and not significant (*p* value was 0.581). This reveals that RP has a non-positive and significant effect on SCB in model M2. The standardized coefficient from RP to SA(a) was 0.22 and the coefficient from SA to SCB(b) was 0.88, which were both significant at the 0.05 level. The chi-square estimate in the model was 1.834. The RMR value is less than 0.05 and RMSEA value is less than 0.08, which reveals the results are significant. The estimates for GFI, CFI and IFI were all greater than 0.9 and close to 1, which proves the model M2 the paper adopted fit the data well.

(3) Based on Wen et al. [73], we used the bootstrap analysis to carry out the following mediation test. Under the 95% confidence level and 2000 bootstrap samples [69], the results are shown in the Table 10 below. The direct unstandardized coefficient from RP to SA(a) was 0.061 and the Z value was 1.968 (>1.96); bias-corrected lower bounds (0.015) to upper bounds (0.133) did not include 0; percentile lower bounds (0.016) to upper bounds (0.137) did not include 0; all of these proved that the direct coefficient form RP to SA(a) was significant. The direct unstandardized coefficient from SA to SCB(b) was 1.189 and the Z value was 5.857 (>1.96); bias-corrected lower bounds (0.929) to upper bounds (1.783) did not include 0; percentile lower bounds (0.908) to upper bounds (1.685) did not include 0; all of these proved that the direct coefficient form SA to SCB(b) was significant. The indirect unstandardized coefficient from RP to SCB(ab) was 0.072 and the Z value was 1.895 (<1.96, but it can be accepted); bias-corrected lower bounds (0.016) to upper bounds (0.164) did not include 0; percentile lower bounds (0.018) to upper bounds (0.169) did not include 0; all of these proved that the indirect coefficient form RP to SCB(ab) was significant. We can draw the following conclusion: the mediating role of SA was significant. Hypothesis H1, H2 and H4 were confirmed.

However, the direct unstandardized coefficient from RP to SCB(c’) was −0.011 and the Z value was −0.647 (|−0.647| < 1.96); bias-corrected lower bounds (−0.04) to upper bounds (0.031) included 0; percentile lower bounds (−0.046) to upper bounds (0.022) included 0; all of these proved that the coefficient form RP to SCB(c’) was not significant. Hypothesis H3 has been rejected.

According to the above, the direct unstandardized coefficient from RP to SA(a) and the direct unstandardized coefficient from SA to SCB(b) were all significant, but the indirect coefficient form RP to SCB(c’) was not significant. In summary, the SA had complete intermediation between RP and SCB.

### 4.2. Discussion

**Figure 4:**
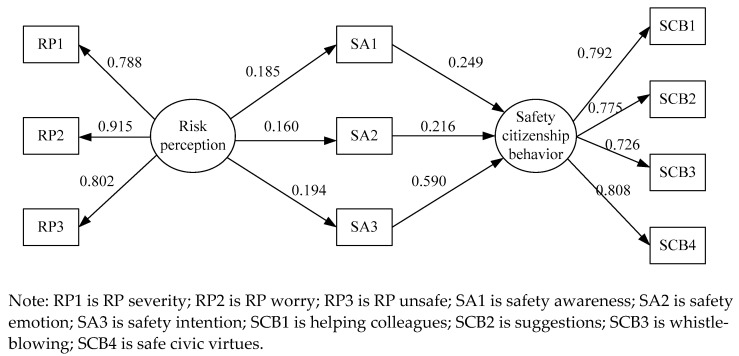
The standardized results of model M3.

In order to further examine the mediating effects of different dimensions of SA on RP and SCB, we constructed model M3. The calculation results are shown in Figure 4 and Table 11 below.

From the correlation coefficients in Figure 4 and Table 11, it was observed that the influence coefficients of RP on RP—severity (RP1), RP—worry (RP2) and RP—unsafe (RP3) were 0.788, 0.915 and 0.802, respectively; the influence coefficients of SCB on helping colleagues (SCB1), suggestions (SCB2), whistleblowing (SCB3) and safe civic virtues (SCB4) were 0.792, 0.775, 0.726 and 0.808, respectively, and they were all significant at the 0.001 level (*p* < 0.001). All these indicate that RP1, RP2 and RP3 play a good role in explaining RP, and SCB1, SCB2, SCB3 and SCB4 can explain SCB well.

From Figure 4 and Table 11, we can get that the mediating effect of safety awareness (SA1) on RP and SCB was 0.046 (0.185 × 0.249); the mediating effect of safety emotion (SA2) on RP and SCB was 0.035 (0.160 × 0.216); the mediating effect of safety intention (SA3) on RP and SCB was 0.114 (0.194 × 0.590), in which SA3 played the most favored mediation effect. The influence coefficients of SA1, SA2 and SA3 on SCB were 0.249, 0.216, and 0.590, respectively, and SA3 has the greatest influence on SCB. This means that SA3 can effectively improve SCB, just as Li [63] proposed that increasing workers’ attention to SA can effectively promote SCB. The influence coefficients of RP on SA1, SA2 and SA3 were 0.185, 0.160 and 0.194, respectively, and RP has the greatest influence on SA3. This further illustrates the mediating role of SA and the importance of SA3. As we already indicated, the dimension ‘‘SA3” emerged as a strong factor in a sense that subway maintenance personnel were first affected by safety intention when they made SCB. Therefore, in order to better improve the SCB of subway maintenance personnel, it is necessary to focus on the enhancement of SA, especially SA3. Of course, the promotion of SA1 and SA2 through RP is equally important.

Secondly, previous studies have shown that demographic factors have an important impact on construction workers’ safety consciousness and SCB [77]. Through the calculation and analysis of the descriptive statistics data, we can see that most of the subway maintenance personnel are men, less than 40 years old, with secondary education experience and more than five years of work experience. They are in the stage of being young and vigorous and full of passion for work, and they are a group of valuable research subjects.

The above data analysis shows that the intermediary role of RP and SCB through SA is closely related. From Table 12, for different genders the SCB of workers was not different, but the RP of males was more sensitive than female workers. The results also show that educational measures for RP should emphasize different aspects for men and women [15].

With aging, the SCB of the workers increases gradually, but the RP is less sensitive. These are also reflected in the length of service. The longer the working experience, the easier it is to make SCB and the worse the sensitivity of RP is.

Previous studies have shown that older workers are twice as likely to have serious/fatal accidents as younger workers [78]. Wetton et al. put forward that if a driver is over 65 years old, his RP test scores will decline with age [79]. Although older employees especially understand their equipment and are creative with its use [80], older employees are more experienced and understand the importance of SCB more; but the increase of experience makes them overlook some potential risks, the sensitivity of the RP falls, their age increases to a certain extent, their physical functions in all aspects decline and their perception of risk also declines.

The worker’s experience is another critical factor that enhances risk perception [81]. Employees with rich work experience and high education show a high level on the perception of safety in the workplace [82]. Highly educated and low-educated employees are more likely to perform SCB. This is because low-educated employees are more compliant with safety regulations, and high-educated employees understand safety regulations better. Therefore, it is possible to enhance the safety management level by strengthening the safety education for employees with low academic qualifications and employees with less experience [83].

In all classifications, the SA of employees is always at a high level, which means that for different categories of employees, even if the SA are the same, the prediction of SCB will be different, and they need to be treated differently. This will be the focus of the next research.

## 5. Conclusions

This article aimed to study the mediating role of SA between RP and SCB in order to find an effective way to improve the quality of metro maintenance personnel SCB with staff. Utilizing data collected from the Subway Company, we obtained 268 valid questionnaires and analyzed the data using SPPS19.0 and AMOS 24.0. The main conclusions from empirical analysis are as follows:

SA plays a complete mediating role between RP and SCB. RP has a significant positive effect on SA [37], and SA significantly promotes SCB [63]. This implies that increasing awareness of RP decreases the probability of incidents, and we can enhance employees’ risk awareness to improve their SA, thereby increasing SCB and effectively achieving the purpose of reducing accidents. The empirical results also show that the RP of subway maintenance personnel has a positive impact on their SA, which greatly affects their SCB. In particular, it needs to be pointed out that the safety intention (SA3) has played an important role in promoting employee SCB. Therefore, in actual work, special attention should be paid to whether the employee’s SA has changed to determine whether the employee’s safety intention is positive or not, which is of great significance for improving the employee’s SCB and reducing accidents. On this basis, combined with the results of the above discussion, we propose the following suggestions:

(1) Employees of different genders have slightly different attitudes and understandings of safety. When choosing training methods, managers should be careful not to generalize completely.
(2) The growth of age and working experience makes employees’ understanding of RP, SA and SCB constantly change. Managers need to fully realize this and select targeted training content and education methods for employees of different ages and working years. In this way, managers can improve their safety level and reduce the accident release rate.
(3) For low-educated employees, managers should do a good job in explaining the various safety regulations to ensure that all employees can correctly understand the meaning and purpose of the safety regulations so that they can fully accept the regulations and reduce the number of violations.
(4) For positions with higher operational risks, employees with more sensitive risk perception and better safety attitudes can be selected.

RP has a close and important relationship with SA and SCB. RP is an important predictor of safety behavior [10] and one of the most important factors to prevent incidents. At the same time, SA plays an important role in the influence of RP on SCB. Therefore, improving the understanding of RP and employee SA has a profound impact on subway safety operations.

Although there is much research on RP, SA and SCB, there is still a lack of research and application in the field of subway maintenance. On the basis of previous studies, this paper studies RP, SA and SCB of subway maintenance personnel, which deepens people’s understanding of the formation mechanism of SCB and enriches the empirical research content of SCB. At the same time, it makes clear the complete intermediary role of SA between RP and SCB, guides the organization to pay attention to the influence of SA on RP and SCB, and puts forward suggestions on safety education, safety training and building a correct safety attitude for employees, which has strong practical significance. However, this study also has some limitations. First of all, the data used in the analysis comes from an online questionnaire survey, and the questionnaire doubts about the items have not been well answered. Therefore, it is recommended that future questionnaires be communicated and answered in advance to reduce the deviation in understanding. Secondly, organizational factors (staff training, working time management, schedule planning, staffing, etc.) were ignored. We plan to introduce organizational factors into the model in the next study.

## Figures and Tables

**Table 1:** Dimension division and abbreviations of risk perception, safety attitude and safety citizenship behavior.

Latent Variable	Dimension	Abbreviations
Risk Perception (RP)	RP—severity	RP1
	RP—worry	RP2
	RP—unsafe	RP3
Safety Attitude (SA)	Safety awareness	SA1
	Safety emotion	SA2
	Safety intention	SA3
Safe Citizenship Behavior (SCB)	Help colleagues	SCB1
	Suggestions	SCB2
	Whistleblowing	SCB3
	Safe Civic Virtues	SCB4

**Table 2:** Basic information of samples.

Item	Classification	Number of People	Proportion
Gender	Male	196	73.1%
	Female	72	26.9%
Age	20~25	52	19.4%
	26~30	56	20.9%
	31~40	130	48.5%
	41~50	26	9.7%
	50 and up	4	1.5%
Years of service	Below 1	16	6.0%
	1~3	29	10.9%
	3~5	35	13.1%
	6~10	77	28.7%
	Above 10	111	41.4%
Educational background	High school and below	25	9.3%
	College	143	53.4%
	Undergraduate	96	35.8%
	Master degree and above	4	1.5%

**Table 3:** KMO and Bartlett Test of Sphericity.

All scale data	KMO measure of sampling adequacy	0.961
	Approximate chi-square	58,271.158
	Freedom	2346
	Significant	0.000
RP	KMO measure of sampling adequacy	0.958
	Approximate chi-square	18,761.958
	Freedom	231
	Significant	0.000
SA	KMO measure of sampling adequacy	0.909
	Approximate chi-square	7509.345
	Freedom	91
	Significant	0.000
SCB	KMO measure of sampling adequacy	0.966
	Approximate chi-square	29,743.669
	Freedom	528
	Significant	0.000

**Table 4:** Results of reliability analysis of each dimension of the questionnaire.

Variable	Test Item	Item Deleted Scale Mean	Scale Variance Value of Item Deleted	Corrected Item Total Correlation (CITC)	Cronbach’s Alpha Value of Item Deleted	Cronbach’s Alpha
RP	RP1	12.467	5.537	0.739	0.836	0.866
	RP2	12.636	6.133	0.799	0.760	
	RP3	12.260	7.551	0.738	0.835	
SA	SA1	13.489	0.829	0.516	0.704	0.729
	SA2	13.377	0.838	0.576	0.612	
	SA3	13.308	1.099	0.618	0.617	
SCB	SCB1	18.723	6.071	0.731	0.859	0.872
	SCB2	19.250	4.633	0.783	0.813	
	SCB3	19.212	4.169	0.739	0.847	
	SCB4	19.033	4.906	0.763	0.823	

Note: RP is risk perception; SA is safety attitude; SCB is safety citizenship behavior; RP1 is RP—severity; RP2 is RP—worry; RP3 is RP—unsafe; SA1 is safety awareness; SA2 is safety emotion; SA3 is safety intention; SCB1 is helping colleagues; SCB2 is suggestions; SCB3 is whistleblowing; SCB4 is safe civic virtues.

**Table 5:** Parameter significance estimation and convergence validity of the measurement model.

Construct	Item	Significance Estimation				Factor Loading	Topic Reliability	Convergent Validity	Composite Reliability
		Un-Std	S.E.	t-Value	*p*	Std	SMC	AVE	CR
RP	RP1	1				0.799	0.638	0.704	0.877
	RP2	0.993	0.067	14.874	***	0.906	0.821		
	RP3	0.734	0.052	14.097	***	0.807	0.652		
SA	SA1	1				0.608	0.37	0.509	0.754
	SA2	1.138	0.142	8.026	***	0.734	0.539		
	SA3	0.862	0.109	7.87	***	0.786	0.617		
SCB	SCB1	1				0.793	0.629	0.667	0.889
	SCB2	1.687	0.114	14.806	***	0.85	0.723		
	SCB3	1.851	0.135	13.681	***	0.793	0.628		
	SCB4	1.545	0.107	14.418	***	0.829	0.688		

Note: RP is risk perception; SA is safety attitude; SCB is safety citizenship behavior; RP1 is RP—severity; RP2 is RP—worry; RP3 is RP—unsafe; SA1 is safety awareness; SA2 is safety emotion; SA3 is safety intention; SCB1 is help colleagues; SCB2 is suggestions; SCB3 is whistleblowing; SCB4 is safe civic virtues; Un-std is unstandardized estimate. S.E. is standard error. *p* is significant. *** At the 0.001 level, the output is significant. Std is standardized estimate. SMC is squared multiple correlations. AVE is average variance extraction. CR is composite reliability.

**Table 6:** The testing results of discriminant validity among RP, SA and SCB.

	SCB	SA	RP	AVE	Square Root of AVE
SCB	1			0.667	0.817
SA	0.876	1		0.509	0.713
RP	0.164	0.217	1	0.704	0.839

Note: RP is risk perception; SA is safety attitude; SCB is safety citizenship behavior; AVE is average variance extraction.

**Table 7:** Overall goodness-of-fit statistics of the confirmatory factor analysis performed.

Goodness-of-Fit Statistics	χ^2^/df	RMR	GFI	IFI	CFI	RMSEA	Reference
Recommended values	1~3	<0.05	>0.9	>0.9	>0.9	<0.08	[71,72,73,74,75,76]
Better value	close to 0	close to 0	close to 1	close to 1	close to 1	close to 0	

Note: χ^2^/df is the chi-square divided by degrees of freedom; RMR is root-mean-square residual; RMSEA is root-mean-square error of approximation; GFI is goodness-of-fit index, IFI is incremental fit index. CFI is comparative fit index.

**Table 8:** The fitting result of model M1.

χ^2^/df	RMR	GFI	IFI	CFI	RMSEA
1.503	0.043	0.981	0.994	0.994	0.043

Note: χ^2^/df is the chi-square divided by degrees of freedom; RMR is root-mean-square residual; RMSEA is root-mean-square error of approximation; GFI is goodness-of-fit index, IFI is incremental fit index. CFI is comparative fit index.

**Table 9:** The fitting result of model M2.

χ^2^/df	RMR	GFI	IFI	CFI	RMSEA
1.834	0.036	0.959	0.981	0.981	0.056

Note: χ^2^/df is the chi-square divided by degrees of freedom; RMR is root-mean-square residual; RMSEA is root-mean-square error of approximation; GFI is goodness-of-fit index, IFI is incremental fit index. CFI is comparative fit index.

**Table 10:** The results of the mediation effect of M2.

Item	Std	Unstd	Product of Coefficient		Bias-Corrected 95% CI		Percentile 95% CI	
			S.E.	Z	Lower	Upper	Lower	Upper
RP->SA(a)	0.217	0.061	0.031	1.968	0.015	0.133	0.016	0.137
SA->SCB(b)	0.882	1.189	0.203	5.857	0.929	1.783	0.908	1.685
RP->SCB(c’) (direct coefficient)	−0.028	−0.011	0.017	−0.647	−0.04	0.031	−0.046	0.022
Indirect coefficient (ab)	0.192	0.072	0.038	1.895	0.016	0.164	0.018	0.169

Note: 2000 bootstrap samples; Std is standardized point estimate; Unstd is unstandardized point estimate. RP is risk perception; SA is safety attitude; SCB is safety citizenship behavior. S.E. is standard error. Z is the value of z test. Lower and Upper are the upper and lower limits of the value.

**Table 11:** Path coefficient and significance of M3.

Path			Unstd	S.E.	C.R.	*p*	Std
SA1	<---	RP	0.128	0.044	2.886	0.004	0.185
SA2	<---	RP	0.104	0.042	2.489	0.013	0.160
SA3	<---	RP	0.089	0.03	3.021	0.003	0.194
SCB	<---	SA1	0.161	0.034	4.799	***	0.249
SCB	<---	SA2	0.148	0.035	4.192	***	0.216
SCB	<---	SA3	0.571	0.055	10.299	***	0.59
RP3	<---	RP	1				0.802
RP2	<---	RP	1.374	0.091	15.097	***	0.915
RP1	<---	RP	1.352	0.097	13.887	***	0.788
SCB1	<---	SCB	1				0.792
SCB2	<---	SCB	1.546	0.119	12.99	***	0.775
SCB3	<---	SCB	1.722	0.143	12.061	***	0.726
SCB4	<---	SCB	1.503	0.111	13.582	***	0.808

Note: RP is risk perception; SA is safety attitude; SCB is safety citizenship behavior; RP1 is RP—severity; RP2 is RP—worry; RP3 is RP—unsafe; SA1 is safety awareness; SA2 is safety emotion; SA3 is safety intention; SCB1 is helping colleagues; SCB2 is suggestions; SCB3 is whistleblowing; SCB4 is safe civic virtues; Unstd is unstandardized estimate. S.E. is standard error. *p* is significant. *** At the 0.001 level, the output is significant. Std is standardized estimate. C.R. is critical ratio; it has the same mean of t-value.

**Table 12:** Average score of RP, SA and SCB.

Item	Classification	Average Score of RP	Average Score of SA	Average Score of SCB
Gender	Male	6.26	6.67	4.69
	Female	6.15	6.77	4.69
Age	20~25	6.58	6.73	4.65
	26~30	6.12	6.54	4.58
	31~40	6.23	6.74	4.74
	41~50	5.79	6.74	4.73
	50 and up	5.84	6.66	4.66
Years of service	Below 1	6.48	6.61	4.46
	1~3	6.55	6.82	4.59
	3~5	6.16	6.61	4.59
	6~10	6.09	6.62	4.64
	Above 10	6.23	6.76	4.81
Educational background	High school and below	6.16	6.73	4.83
	College	6.33	6.71	4.64
	Undergraduate	6.12	6.66	4.72
	Master degree and above	5.48	6.77	4.82

## Appendix A

The original version of this questionnaire was in Chinese and was translated into English for presentation here. All the items were measured using a seven-point Likert scale.

**Table A1:** Contents of scales.

Risk perception scale	RP Severity: If you encounter the following work incidents or situations, how serious do you think the potential negative consequences are?	When working on electrical equipment, accidental power transmission
		Accidentally touch the bare part of the conductor without electrical inspection
		Hit by objects falling from a height
		Falling from a height
		Do not wear protective equipment and perform dangerous operations such as welding, power distribution, carrying heavy objects, painting chemicals, etc.
		The vehicle slips due to unstable parking or no braking (or slipping occurs)
		Tripped over materials, equipment, grounding wires, etc. stored in the workplace
	RP Worry: If you encounter the following work incidents or situations, how will you worry about potential negative effects?	No electrical inspection and grounding before work
		Perform strictly prohibited behaviors at work (such as calling, smoking, screaming, chasing, etc.)
		Working at high places (such as working on the top of the train or on the scaffolding at an equal distance above 2 m from the falling datum)
		Passing (passing through) lifting movement route
		The ground in the workplace or walking route is uneven or has obstacles
		Limited space operation
		Work in a workplace without adequate lighting
		Do not wear protective equipment (such as earmuffs, masks, gloves, etc.) during work
	RP Insecurity: If you encounter the following work incidents or situations, how insecure would you feel about potential negative consequences?	Start working directly when the electrical equipment is not connected to the grounding wire
		No warning signs and isolation belts are placed on high-altitude dangerous goods at the scene
		When working at height, the safety line is not fastened
		The workplace is full of debris
		Working in a noisy environment
		Straying into another person’s workplace
		Dimly lit workplace
Safety Attitude Scale	Safety Awareness	I think safety accidents at work can be prevented
		I think wearing safety protective gear can prevent accidents
		I think using safe equipment can improve my job safety
		I think a clean and tidy workplace can help reduce the occurrence of safety incidents
		I think safety regulations must be strictly followed
		I think strict and standardized safety management will help reduce accidents
	Safety Emotion	I am happy to accept others’ instructions on my work safety
		I am happy to accept help from others in my work safety
		I am willing to wear safety protection equipment for work
	Safety Intention	If I see a warning sign ahead, I will go around
		Before I start working, I will put on safety shoes and a helmet
		I will carefully understand how to use protective gear and wear it at work
		Before starting work, I tend to check equipment and facilities for safety hazards
		If there is a problem with the device, I will stop working immediately
Safety Citizenship Behavior Scale	Help Colleagues	I will take the initiative to help workers familiarize themselves with the environment
		I will take the initiative to help workers learn safe working procedures
		I will take the initiative to help workers learn safe work rules and regulations
		I will take the initiative to help workers understand the responsibilities and obligations related to safety
		I will take the initiative to help workers learn the correct use of safety protective equipment
		I will take the initiative to help workers learn the correct operation process of facilities and equipment
		I will urge workers to wear safety protection equipment neatly before work
		I will urge workers to check whether the facilities and equipment are in good condition before work
		I will urge workers to pay attention to whether the surrounding environment is safe when working
		I will urge the workers to follow the leadership’s task assignment for safety matters
		I will take the initiative to help workers use facilities and equipment safely
		I will take the initiative to help workers eliminate safety hazards around them
		I will take the initiative to help workers stay away from danger
	Suggestions	I will express my opinion on security issues even if others disagree
		During the work process, I will put forward relevant suggestions to improve the status quo of construction safety
		I try to make suggestions for improving the safety of work tasks
		I tried to change the way I work to make work safer
		I will make corresponding safety recommendations for the working environment
		I will propose improvements to the protective gear currently in use
		I will propose improvements to the facilities and equipment currently in use
		I tried to change the safety policies and procedures to make work safer
		I will put forward the unrealistic content in the safety regulations and propose improvement measures
		I will take the initiative to help leaders organize safety training
		I will take the initiative to help leaders collect and sort out the safety issues in work
	Whistleblowing	I will report other people’s violations of safety regulations
		I will report violations of safe work procedures by others
		I will report the behavior of others not wearing safety protection equipment
		I will report other people’s illegal use of the device
		I will report other people’s behaviors that hinder the safety of the work space (for example, random stacking of debris)
		I will report other people’s behavior that they started using without checking the device
	Safe Civic Virtues	I will take the initiative to repair and maintain work facilities and equipment
		I will take the initiative to investigate the safety hazards in the workplace
		I will always pay attention to the debris left on the road and clean it up in time
